# Inhibition of Neoplastic Transformation and Chemically-Induced Skin Hyperplasia in Mice by Traditional Chinese Medicinal Formula Si-Wu-Tang

**DOI:** 10.3390/nu9030300

**Published:** 2017-03-18

**Authors:** Mandy M. Liu, Kevin M. Huang, Steven Yeung, Andy Chang, Suhui Zhang, Nan Mei, Cyrus Parsa, Robert Orlando, Ying Huang

**Affiliations:** 1 Department of Pharmaceutical Sciences, College of Pharmacy, Western University of Health Sciences, Pomona, CA 91766, USA; mmliu@westernu.edu (M.M.L.); huang.2834@buckeyemail.osu.edu (K.M.H.); skyeung@westernu.edu (S.Y.); chang.andy.y@gmail.com (A.C.); 2 Department of Pharmacology and Toxicology, Shanghai Institute for Food and Drug Control, Shanghai 201203, China; suhuizhangbc@gmail.com; 3 Division of Genetic and Molecular Toxicology, National Center for Toxicological Research, Jefferson, AR 72079, USA; nan.mei@fda.hhs.gov; 4 Department of Clinical Sciences, College of Osteopathic Medicine, Western University of Health Sciences, Pomona, CA 91766, USA; cparsa@westernu.edu (C.P.); rorlando@beverly.org (R.O.)

**Keywords:** cancer prevention, DMBA, skin cancer, AP-1, NF-κB, SWT, SENCAR mice, EGF, JB6, Ames test

## Abstract

Exploring traditional medicines may lead to the development of low-cost and non-toxic cancer preventive agents. Si-Wu-Tang (SWT), comprising the combination of four herbs, Rehmanniae, Angelica, Chuanxiong, and Paeoniae, is one of the most popular traditional Chinese medicines for women’s diseases. In our previous studies, the antioxidant Nrf2 pathways were strongly induced by SWT in vitro and in vivo. Since Nrf2 activation has been associated with anticarcinogenic effects, the purpose of this study is to evaluate SWT’s activity of cancer prevention. In the Ames test, SWT demonstrated an antimutagenic activity against mutagenicity induced by the chemical carcinogen 7,12-dimethylbenz(a)anthracene (DMBA). In JB6 P+ cells, a non-cancerous murine epidermal model for studying tumor promotion, SWT inhibited epidermal growth factor (EGF)-induced neoplastic transformation. The luciferase reporter gene assays demonstrated that SWT suppressed EGF-induced AP-1 and TNF-α-induced NF-κB activation, which are essential factors involved in skin carcinogenesis. In a DMBA-induced skin hyperplasia assay in ‘Sensitivity to Carcinogenesis’ (SENCAR) mice, both topical and oral SWT inhibited DMBA-induced epidermal hyperplasia, expression of the proliferation marker Proliferating cell nuclear antigen (PCNA), and H-*ras* mutations. These findings demonstrate, for the first time, that SWT prevents tumor promoter and chemical-induced carcinogenesis in vitro and in vivo, partly by inhibiting DNA damage and blocking the activation of AP-1 and NF-κB.

## 1. Introduction

Traditional Chinese herbal medicines provide a rich source for the development of alternative and complimentary medicines for cancer therapy and prevention. Si-Wu-Tang [SWT, Si-Wu decoction (Chinese name), Samultang (Korean name), or Shimotsu-to (Japanese name)], comprising the combination of four herbs: Paeoniae (*Radix paeonia alba*), Angelicae (*Radix angelica Sinensis*), Chuanxiong (*Rhizoma chuanxiong*), and Rehmanniae (*Radix rehmanniae preparata*), is one of the most popular traditional medicines for women’s health [1]. It has been used in Eastern Asia for more than one thousand years and ranks first as the most frequently used Chinese medicines [2]. It is an inexpensive over-the-counter preparation used for the relief of menstrual discomfort, climacteric syndrome, peri- or post-menopausal syndromes and other estrogen-related diseases [1,2,3,4,5]. In previous animal studies, SWT has shown sedative, anti-coagulant, and anti-bacterial activities, as well as a protective effect on radiation-induced bone marrow damage [6,7]. Several in vitro and in vivo studies show a preventive activity of SWT on endometrial carcinogenesis induced by chemical carcinogen sand estrogen [8,9]. However, the mechanisms and bioactive constituents mediating these effects are unknown.

The nuclear factor erythroid 2-related factor 2 (Nrf2), a basic zip (bZIP) transcription factor, is a key molecule that regulates detoxifying and antioxidant genes [10]. The Nrf2 pathway has become a promising molecular target for the chemoprevention of cancer (for review, see [11]). Using DNA microarray and connectivity map-based analysis of the gene expression profiles in the MCF-7 breast cancer cells, our previous studies revealed the potential mechanism of SWT, which involves the activation of Nrf2-regulated antioxidant genes, such as *HMOX1, GCLC, GCLM, SLC7A11*, and *NQO1* [12]. We further provide experimental evidence to show that SWT protected cells against oxidative stress, and enhanced the translocation of Nrf2 into the nucleus in non-cancerous mammary epithelial cells [13]. In a study using healthy Sprague−Dawley rats to evaluate the in vivo pharmacodynamic effect of SWT, short-term oral administration of SWT (1000 mg/kg per day for six consecutive days) caused an increased expression of Nrf2-regulated genes *Hmox1* and *Slc7A11* in the liver [13]. In addition, SWT has been previously reported to have a suppressive effect on estrogen-induced inflammatory enzyme COX-2 [14].

Since carcinogenesis involves multiple abnormal genes/pathways, using an herbal formula, such as SWT, may be superior to agents that target a single molecular event. While there are other natural products known as Nrf2 activators, SWT provides a good option due to multiple mechanisms and its clinical safety record. Therefore, in the present study, we investigated SWT’s effect on mutagenicity and carcinogenesis in several in vitro and in vivo model systems, mainly of skin cancer. We further explored possible mechanisms that may underlie the chemopreventive effects. Based on these experimental data, we predict that the chemopreventive activity of SWT is not limited to skin cancer, but with a broad application for other types of cancer associated with oxidative stress.

## 2. Materials and Methods

### 2.1. Compounds

7,12-dimethylbenz[a]anthracene (DMBA) and 2-nitrofluorene were purchased from Sigma-Aldrich (St. Louis, MO, USA). EGF was purchased from Peprotech (Rocky Hill, NJ, USA) and dissolved in sterile deionized water as 100 µg/mL stock and stored at −20 °C in a freezer.

### 2.2. Preparation of Herbal Extracts

The SWT extract and its component single herb extracts were kindly provided by Dr. Z. Zuo at the School of Pharmacy, Chinese University of Hong Kong. These products were manufactured under Good Manufacturing Practice (GMP) conditions at the Hong Kong Institute of Biotechnology (Hong Kong, China) according to the protocol described in Chinese Pharmacopoeia 2005 [15] with slight modification. Therefore, these products are named as ‘Chinese University-SWT’ (‘CU-SWT’), and the single herb extracts named as CU-Angelicae, CU-Chuanxiong, CU-Paeoniae, and CU-Rehmanniae. The formulae were made in solid dosages (powder form). The sources and ratio of the herbal components, as well as chemical fingerprints of the SWT product used in this study, have been described before [1]. In brief, Angelicae and Chuanxiong (2.5 kg each) were soaked in water for 0.5 h followed by steam distillation, after which the volatile oil phase, aqueous phase, and the solid residues were collected. To 2.5 L volatile oil, 125 g hydroxypropyl-beta-cyclodextrin was added, and the vessel was covered to protect from light during mixing by magnetic stirring at room temperature for one hour. To the residue of the herbs, Paeoniae and Rehmanniae (2.5 kg each) were added and decocted with boiling water three times, successively. All of the aqueous phase solutions from each decoction were combined. The aqueous and oil phase extracts were spray dried and freeze dried, respectively, to produce the corresponding powders before combining to obtain the final product. Previous study has developed methods to identify markers in SWT products [1]. Five compounds are detectable in all batches of SWT extracts, including ‘CU-SWT’ used in this study. For the compounds paeoniflorin, ferulic acid, gallic acid, z-Liguistilide, and senkyunolide A, the CU-SWT extract contains 0.82%, 0.076%, 0.084%, 0.14% and 0.0082%, respectively. The preparation of single herb products was also carried out according to the aforementioned procedure. The herbal solutions were prepared fresh from powder right before the experiment in medium and sonicated for 30 min. The powders were only partially dissolved. Without centrifugation, the whole solutions were added to the cell culture experiments described below (Section 2.7, Section 2.8 and Section 2.9).

### 2.3. Bacterial Strains and Growth Conditions

The *Salmonella typhimurium* strain TA100 is a histidine-requiring mutant, as previously described by Maron and Ames [16], and was purchased from MOLTOX (Boone, NC, USA) and stored at −80 °C. Tests of histidine requirements, as well as the genotypes of *rfa*, *uvr*B mutation, and R factor, were carried out to confirm the genotypes of TA100 (data not shown). TA100 contains the base-pair substitution mutation hisG46. It was grown for 10 h with gentle shaking in nutrient broth No. 2 (Oxoid, Hampshire, UK) at 37 °C.

### 2.4. Mutagenicity Testing

The test was conducted based on the plates incorporation method [16], using TA100 with or without exogenous metabolic activation system S9, purchased from MOLTOX (Boone, NC, USA). The Ames test without S9 can only detect direct mutagens, while with S9 metabolic activation allows the detection of indirect mutagens, often caused by conjugation reactions of metabolic oxidation systems [16]. Various concentrations of SWT were added to the top agar (2.5 mL), supplemented with 0.5 mM l-histidine and 0.5 mM d-biotine, mixed with 100 μL of bacterial culture (approximately 1.4–1.6 × 10^8^ cells), and then poured onto a minimal glucose agar plate and incubated at 37 °C for 48 h before counting the his+ revertant colonies. Five hundred microliters of S9 mixture were added into top agar (2.0 mL) to test the influence of metabolic activation. Dimethyl sulfoxide (DMSO) was added into the top agar as a negative control group. The number of revertants per plate in the negative control and positive control groups were within the normal limits found in our laboratory. The data were collected in mean ± SD in three plates (*n* = 3).

### 2.5. Antimutagenicity Testing

Using a procedure the same as the mutagenicity testing was employed to determine the effect of SWT on mutagenicity induced by 2-nitrofluorene or DMBA. The test agents, together with S9 mix (500 μL), mutagens (100 μL), SWT (100 μL), and 100 μL of bacteria culture, were added into 2 mL or 2.5 mL of top agar. The plates were incubated at 37 °C for 48 h and then the his+ revertant colonies were counted. The inhibition rate of mutagenicity (%) was calculated using the following equation:

Inhibition rate (%) = 1 − (A/B) × 100%
(1)

where A is the number of revertants per plate in the presence of direct or indirect mutagen and SWT, and B is the number of revertants per plate in positive control group.

### 2.6. Cell Culture

JB6 CI 41-5a (JB6 P+), sensitive to the promotion of transformation mouse epidermal cells, were purchased from American Type Culture Collection (ATCC, Manassas, VA, USA). JB6 P+ were maintained in Eagle’s minimum essential medium (EMEM) containing 4% heat-inactivated fetal bovine serum and 1% penicillin/streptomycin. The HEK-293 cells and MCF-7 cells were obtained from ATCC, cultured in DMEM, supplemented with 10% FBS and 1% penicillin-streptomycin. All cells from cell culture experiments were incubated at 37 °C in 5% CO_2_/95% air.

### 2.7. Anchorage-Independent Growth Assay in Soft Agar

In a 96-well tissue culture plate, 2000 JB6 P+ cells per well were mixed with 0.33% agar suspended on top of a layer of 0.5% agar. Epidermal growth factor (EGF) (10 ng/mL) was used to promote the anchorage-independent growth of JB6 cells. Various concentrations of SWT were added together with EGF into the top and bottom layers of the agar. Plates were incubated at 37 °C for 7–10 days. Colonies with greater than ten cells were counted and images were taken using EVOS Cell Imaging Systems (Thermo Fisher Scientific, Waltham, MA, USA).

### 2.8. Cell Proliferation Assay

Ninety six-well plates were seeded with 3000–4000 JB6 P+ cells per well and allowed to attach overnight. Cells were treated with test compounds for 72 h and incubated at 37 °C in 5% CO_2_/95% air. Cell viability was determined using Sulforhodamine B (SRB) assay (Sigma) according to the manufacturer’s protocol.

### 2.9. Luciferase Reporter Gene Assay

HEK-293 or MCF-7 cells were transfected with pGL4.22-AP1 (gift from Dr. D. Sanchez) or pGL4.22-NF-κB (Promega, Madison, WI, USA), mixed with pRL-TK-luc (Promega) at a 40:1 ratio using FuGENE HD Transfection Reagent (Roche, Indianapolis, IN, USA) according to the manufacturer’s instructions. Twenty-four hours after transfection, the cells were exposed to test agents for another 24 h (for AP-1) or 5 h (for NF-κB). Cell lysates were used for determining luciferase activities of both firefly and renilla by the dual luciferase reporter gene assay (Promega). Firefly luciferase activity was normalized to renilla luciferase activity. The experiment was carried out in triplicate and expressed as the mean ± SD.

### 2.10. Model of Chemically-Induced Murine Skin Hyperplasia

All animal studies were carried out in strict accordance with the recommendations in the Guide for the Care and Use of Laboratory Animals of the National Institutes of Health, and approved by the Western University of Health Sciences Institutional Animal Care and Use Committees. Five-week-old female SENCAR mice (National Cancer Institute, Frederick, MD, USA) were divided into six groups (*n* = 6 or 8) and the backs of mice shaved. At seven weeks of age, 100 nmol DMBA dissolved in 200 µL acetone was applied topically twice weekly for four weeks. SWT treatment started when mice were five weeks of age, twice weekly, topically in two doses (0.64 and 1.28 mg/mL in 200 µL acetone) 30 min before DMBA exposure, or orally by gavage in two doses (200 and 1000 mg/kg in 1% methyl cellulose in PBS) 2 h before DMBA exposure. Two days after the last treatment mice were sacrificed, and samples of skin were excised and fixed immediately in formalin and embedded in paraffin blocks. The embedded tissues were cut into 3-micron thick sections and stained with H&E to determine the morphology. The images were obtained by EVOS; the epidermal thickness was measured using a Nikon Live-Cell Imaging system (Melville, NY, USA).

### 2.11. Immunohistochemistry (IHC) Analysis

Paraffin-embedded sections were baked at 60 °C for one hour and deparaffinized in a xylene solution and rehydrated through a graded series of ethanol. The antigen was retrieved using a citrate buffer (pH 6.0) for 20 min at 95 °C. Briefly, sections were blocked by 10% normal goat serum for 2 h followed by overnight incubation at 4 °C with 1:1000 dilution of the proliferation cell nuclear antigen (PCNA; Cell Signaling Technologies, Danvers, MA, USA) primary antibody. Sections were then incubated for 2 h with 1:5000 dilution of an horseradish peroxidase (HRP) secondary antibody, followed by 5-min incubation with DAB substrate (Vector labs; Burlingame, CA, USA) and counterstained with Mayer’s hematoxylin.

### 2.12. Competitive Allele-Specific TaqMan PCR (castPCR) Assay

Genomic DNA was isolated from frozen skin tissues by DNAzol (MRC, Inc., Charleston, WV, USA) according to the protocol provided by the manufacturer. CastPCR was performed with TaqMan Mutation Detection Assay designed to detect CAA → CTA transversion in codon 61 of the mouse H-*ras* gene (Applied Biosystems by Life Technologies, Foster City, CA, USA) following the manufacturer’s instruction. The castPCR was run on a GeneAmp 7300 Sequence Detection system (Applied Biosystems, Foster City, CA, USA) using the universal mutation detection thermal-cycling protocol. The mutational status of a sample was determined by calculating the ΔCt value between the mutant allele assay and wild-type allele assay to obtain the percent mutation according to manufacturer’s instruction.

### 2.13. Statistical Analysis

All in vitro data are expressed as the mean ± standard deviation of three independent experiments under the same experimental conditions, and in vivo data are expressed as the mean ± standard error. The one-way ANOVA test was used to analyze the results and a *p* value < 0.05 was denoted as significant. Variants of statistical analysis are otherwise stated in figure legends.

## 3. Results

### 3.1. Mutagenic and Antimutagenic Activity of SWT

SWT was firstly evaluated for mutagenic and antimutagenic activity using the Ames test, conducted using the *S. typhimurium* TA100 bacterial strain in the presence or absence of the metabolic activator S9 system. Two reference mutagens were used as positive controls: the direct mutagen 2-nitrofluorene (−S9), and the indirect mutagen DMBA (+S9). Both of them (10 µg/plate) caused a strong mutagenic effect (Table 1: 27 and 11-fold increases in the number of revertant colonies in comparison with the negative controls for direct and indirect mutagens, respectively). However, no cytotoxic (i.e., normal bacterial lawn) and no mutagenic (i.e., similarly numbers of revertant colonies as negative controls) activities were observed for all doses tested for SWT up to 5 mg per place in the presence or absence of the S9 system. This result indicates that SWT within the concentration range tested may not be mutagenic nor be metabolized into mutagens. Since mutagenicity is correlated with carcinogenicity [17], this result suggests that it is potentially safe to use SWT (up to 5 mg/plate) as a preventive agent for healthy individuals even at higher doses.

When combined with mutagens, SWT at doses <5 mg/plate did not show an effect of inhibition on mutagenicity induced by the direct mutagen 2-nitrofluorene (−S9) (Table 1). However, significant antimutagenic activity (*p* < 0.05) was observed in doses of 5 mg/plate against the mutagenicity induced by DMBA (Table 1). Since antimutagenic agents can possibly also be anticarcinogens, these results indicate that SWT may protect cells against harmful effects resulting from the indirect mutagen DMBA. These results are consistent with the results obtained from the in vivo study described below, which uses DMBA as a carcinogen.

### 3.2. Effects of SWT on EGF-Induced Neoplastic Transformation of JB6 P+ Cells

**Figure 1:**
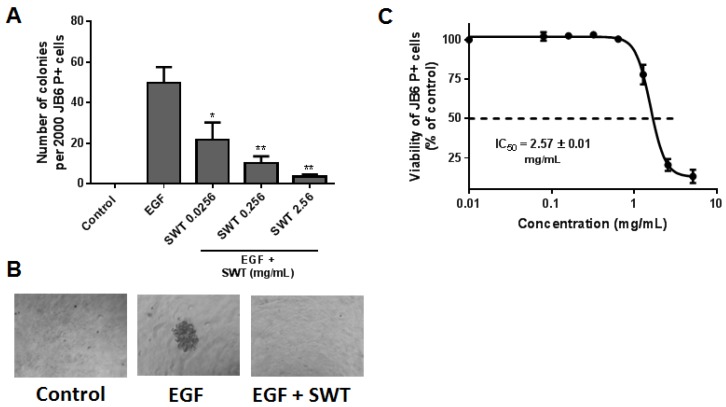
Effects of SWT extract on EGF-mediated neoplastic cell transformation and colony formation in JB6 P+ cells. (**A**) Soft agar assay data for JB6 P+ cells treated with EGF (10 ng/mL) alone or together with SWT at three concentrations. Colonies greater than ten cells were counted manually under a microscope. Data represents a mean ± standard deviation (*n* = 6-12). *: *p* < 0.05; **: *p* < 0.01, compared to EGF only group; (**B**) representative images of colonies during the soft agar assay after a 10 day incubation period; and (**C**) the cytotoxic effects of SWT on JB6 P+ cells were examined using an SRB assay. Results are expressed as percentage of viability versus control cells with no drug treatment.

We next examined the effects of SWT on EGF-mediated neoplastic transformation of the mouse epidermal JB6 P+ cell line, which is a well-characterized model for studying cellular response to various tumor promoters [18]. Since EGF and its receptor (EGFR) have been reported as an important signaling pathway leading to cancer, EGF was used to promote JB6 transformation. When treated with EGF, the transformation sensitive P+ cells acquired anchorage-independent growth, i.e., colony formation in soft agar. Treatment with SWT resulted in drastic inhibition of EGF-induced transformation and colony formation in a dose-dependent manner compared to the number of colonies induced by EGF alone (Figure 1A). Representative images of colonies in soft agar are shown in Figure 1B.

As the soft agar assay is dependent on cell viability, we conducted sulforhodamine B (SRB) colorimetric assay for evaluating the effects of SWT on cell growth and cytotoxicity. Treatment of JB6 P+ cells with SWT was non-toxic at concentrations lower than 0.3 mg/mL, while higher concentrations caused growth inhibition (IC_50_ = 2.57 ± 0.01 mg/mL) (Figure 1C). Thus, SWT at the non-toxic concentrations 0.0256 and 0.256 mg/mL inhibited EGF-mediated colony formation due to a direct effect on tumor promotion (Figure 1A), while the colony inhibitory effect of SWT at 2.56 mg/mL may be attributed to a mixed activity of anti-promotion and cytotoxicity. Since the JB6 P+ transformation assay has a positive predictive value for in vivo efficacy of chemopreventive agents [19], this result indicates that SWT may have chemopreventive activity at non-toxic concentrations and cytotoxic action at higher concentrations.

**Figure 2:**
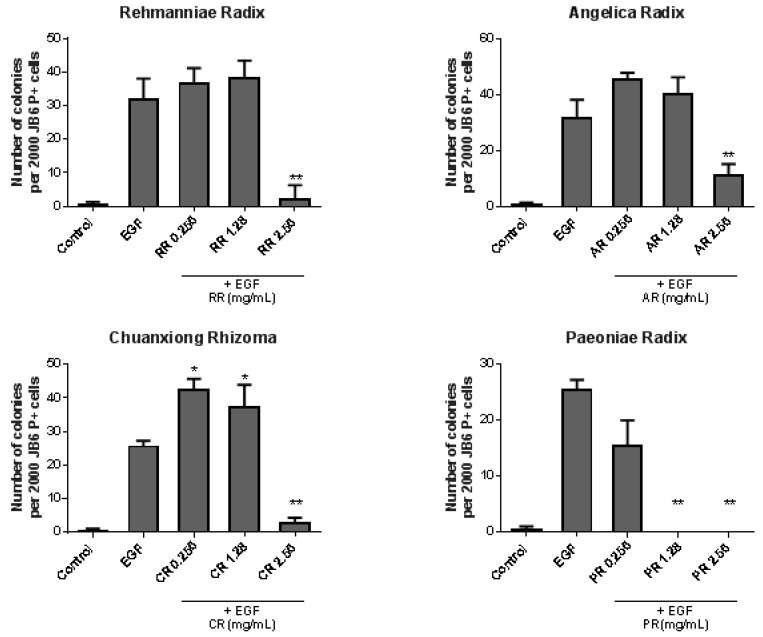
Effects of four single herbal components of SWT on EGF-induced malignant transformation of JB6 P+ cells. Soft agar assay was conducted on JB6 P+ cells treated with EGF (10 ng/mL) and/or SWT herbal components at three concentrations. Colonies greater than ten cells were counted manually under a microscope. Data represents a mean ± standard deviation (*n* = 6-12). *: *p* < 0.05; **: *p* < 0.01, compared to EGF only group.

The soft agar assay experiment was also conducted to examine whether the four herbal components of SWT can also inhibit EGF-induced colony formation in JB6 P+ cells. The results showed that all extracts possess strong and significant chemopreventive activity at higher concentration (2.56 mg/mL) (Figure 2). Among the four herbs, Paeoniae (PR) showed the highest potency because, at 1.28 mg/mL, it completely blocked the colony formation. However, this effect may be due to cytotoxicity. SRB assay showed that the IC_50_ for Paeoniae in JB6 P+ cells was 0.5 mg/mL, while IC_50_ of the other three components was higher than 2.56 mg/mL (data not shown). Therefore, PR may contain the most toxic constituents of SWT. At lower concentrations (0.256 and 1.28 mg/mL), the other three extracts slightly increased EGF-induced colony formation, although such effect is significant only for Chuanxiong (CR). Unlike SWT (Figure 1), none of the four herbs showed significant inhibitory effect at a concentration of 0.256 mg/mL. Thus, SWT showed superior activity against tumor promotion which cannot be simply attributed to an additive effect of mixing four herbs. A synergistic mechanism is possible leading to a unique SWT formula which is not only more effective but also less toxic.

### 3.3. SWT on EGF-Induced AP-1 Activation

**Figure 3:**
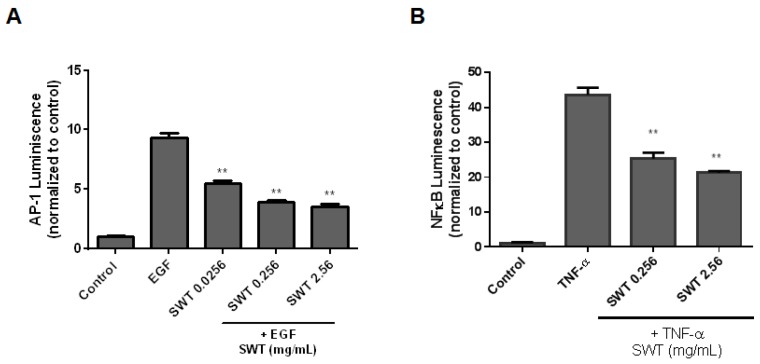
Effects of SWT on AP-1 and NF-κB activation. (**A**) Luciferase assay using HEK-293 cells co-transfected with a plasmid containing an AP-1-luciferase reporter gene (pGL4.22-AP1) and a plasmid encoding renilla luciferase (pGL4.74). The transfected cells were treated with EGF (10 ng/mL) and SWT for 24 h prior to measurement of firefly and renilla luciferase activities using the dual luciferase reporter gene assay; and (**B**) luciferase assay using MCF-7 cells co-transfected with a plasmid containing an NF-κB-luciferase reporter gene (pGL4.22-NF-κB) and a plasmid encoding renilla luciferase (pGL4.74). The transfected cells were treated with TNF-α (20 ng/mL) and SWT for 5 h prior to measurement of firefly and renilla luciferase activities using the dual luciferase reporter gene assay. **: *p* < 0.01.

AP-1 is a major transcription factor involved in EGF-induced transformation of JB6 P+ cells [20]. To further explore SWT’s possible mechanisms of action, its effect on EGF-mediated activation of AP-1 was evaluated. The HEK-293 cells were transfected with AP-1 firefly luciferase reporter and the renilla luciferase control reporter. The transfected cells were treated with vehicle (control), SWT (0.025, 0.256, and 2.56 mg/mL), followed by a 24-h co-incubation with EGF (10 ng/mL). As can been seen in Figure 3A, SWT significantly inhibited EGF-mediated AP-1 activity in a dose-dependent manner (*p* < 0.01). These results indicated that inhibition of AP-1 activation by SWT may partly explain the mechanism underlying the inhibitory activity against EGF-induced cell transformation. Since variety of agents that inhibit AP-1 has been reported as mechanism(s) of chemoprevention [20], and another possible target for SWT is AP-1.

### 3.4. Effects of SWT on TNF-α-Induced NF-κB Activation

To evaluate the effect of SWT on inducible NF-κB activation, the breast cancer MCF-7 cells were transfected with NF-κB-luc reporter construct and a plasmid encoding renilla luciferase. The transfected cells were treated with vehicle (control), SWT (0.256 and 2.56 mg/mL), followed by a 5-h co-incubation with TNF-α (20 ng/mL). NF-κB activity was measured by a dual luciferase reporter gene assay. TNF-α stimulated NF-κB activity, and this activity was inhibited by SWT (Figure 3B). Since previous findings support a role for NF-κB in promoting carcinogenesis [21], our results suggest that therapeutic targeting of the NF-κB by SWT might be one of the mechanisms of SWT in prevention of cancer.

### 3.5. Effects of SWT on DMBA-Induced Skin Hyperplasia in SENCAR Mice

**Figure 4:**
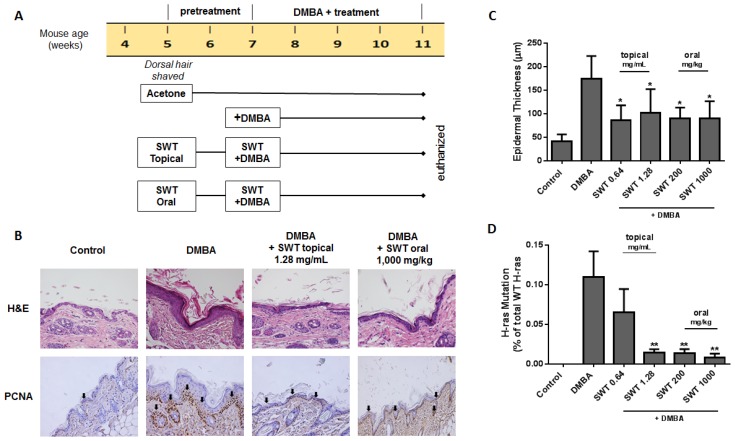
Effects of SWT on DMBA-induced hyperplasia and mutation of H-*ras* in SENCAR mice. (**A**) Experimental design; (**B**) microphotographs of H&E staining, and immunohistochemistry of PCNA to depict DMBA-induced skin hyperplasia and cell proliferation activity. Black arrow: positive PCNA cells in stratum basale of epidermis; (**C**) induction of epidermal thickness with DMBA and the effect of SWT. Data represents the mean ± standard error from repeated measurements of one sample at 20 locations along tissue; and (**D**) the percentage of H-*ras* codon 61 mutations (CAA → CTA) detected using castPCR. Statistical analysis was performed by ANOVA with Dunnett’s multiple comparisons post-hoc test. *: *p* < 0.05; **: *p* < 0.01, compared to DMBA only group (*n* = 6–8).

To determine the in vivo chemopreventive activity of SWT, a DMBA-induced skin hyperplasia assay in SENCAR mice was utilized [22]. The experimental design is shown in Figure 4A. Epidermal hyperplasia was induced by topical treatments with DMBA for four weeks. The SWT treatments included two topical doses (0.64 and 1.28 mg/mL) and two oral doses (200 and 1000 mg/kg), beginning two weeks before the first dose of DMBA. During this study period, the treatments did not cause animal death; SWT did not cause any visible sign of toxicity or ill health, nor have any significant effect on body weight in mice (data not shown). Representative samples of the epidermis (H&E staining) are shown in Figure 4B. Epidermal thickness was measured 20 times at various locations along the epidermis and averaged to obtain a single sample’s value (Figure 4C). DMBA treatment increased the average epidermal thickness by 4.2-fold from 41.47 ± 14.88 μm in controls to 174.68 ± 47.57 μm in the DMBA group. Both topical and oral treatments resulted in significantly decreased epidermal thickness compared with the DMBA-treated group without obvious dose-dependent effect (Figure 4C). Analogous to the H&E data, immunohistochemical analysis indicated an increased expression of the proliferating cell nuclear antigen (PCNA) in the stratum basale of epidermis after DMBA treatment, while SWT treatment resulted in reducing the number of PCNA positive cells (Figure 4B).

One of the key events in tumor initiation in mouse skin is the mutation of H-*ras* at codon 61 (CAA → CTA) [23]. The Mutation Detection Assays based on Competitive Allele-Specific TaqMan PCR (castPCR) technology was used to assess H-*ras* mutations in mouse skin DNA samples. The castPCR is a highly specific and sensitive method for detecting and quantitating rare mutations in a sample that contains large amounts of normal, wild-type genomic DNA [24]. The results showed that the control mice had no mutations, while the DMBA treatment group harbored an average of 0.1% of the mutations (Figure 4D). The topical treatment by SWT dose-dependently inhibited the mutations induced by DMBA. The high dose topical group resulted in 7.6-fold reduction of the mutation induced by DMBA. For the oral treatment groups, low and high dose treatments resulted in a similar inhibitory effect as the high topical dose group, indicating oral route may also be effective for preventing the oncogenic mutations. These results indicate that SWT effectively blocked skin hyperplasia and tumor-initiating mutations induced by chemical carcinogens in vivo.

## 4. Discussion

Many risk factors associated with skin carcinogenesis, including ultraviolet radiation and environmental pollutants, damage epidermal cells through the generation of high levels of reactive oxygen species (ROS) [25]. The formation of ROS can be both an initiator and promoter for skin cancer. Protecting normal cells from the attack of ROS by increasing the cellular detoxifying and antioxidant machinery is a promising strategy to prevent cancer [26]. We previously reported that SWT activated Nrf2-regulated genes, which play an important role in cellular detoxification [12]. Therefore, in the present study we evaluated the cancer chemopreventive efficacy of SWT in vitro and in vivo. The mouse epidermal JB6 P+ cell line is a well-characterized model for studying neoplastic transformation in response to various tumor promoters and for screening chemopreventive agents [18]. EGF was used to promote transformation, because EGF and its receptor (EGFR) have been found as an important signaling pathway leading to cancer, including skin cancer [27]. Under EGF exposure, the transformation sensitive JB6 P+ cells acquired anchorage-independent growth and tumorigenicity. We found that SWT inhibited anchorage-independent growth of JB6 P+ cells on soft agar induced by EGF (Figure 1), which was further confirmed in vivo using DMBA-induced mouse skin hyperplasia assay (Figure 4). An Ames test was used to test the mutagenic and antimutagenic activities of SWT. Although SWT itself showed an absence of any mutagenic activity, at the dose of 5 mg/plate it exhibited significant inhibition on the reversion of *S. typhimurium* TA100 induced by the indirect mutagen DMBA in the presence of S9. This result indicated that SWT may protect cells against carcinogen-induced DNA damage. The same chemical carcinogen, DMBA, was used to induce skin hyperplasia and oncogenic mutation in mice, and our data confirmed SWT’s protective effect in vivo. Although we cannot exclude any direct interaction between SWT components and DMBA, these results demonstrated SWT’s anti-carcinogenic effects in vitro and in vivo. One of the possible targets for SWT is the Nrf2 molecule, which is expressed in all cell types of skin and is a key molecule in skin homeostasis [28]. The role of Nrf2 in skin carcinogenesis has been shown using knockout mice or with pharmacological activators of Nrf2 [28]. Other potential targets for SWT identified in our study are AP-1 and NF-κB, which are transcription factors implicated in EGF-induced skin tumor promotion [20]. Inhibition of AP-1 and NF-κB by a variety of agents has been shown as the major mechanism of chemoprevention [20]. It is likely that the multiple components in SWT trigger the signaling in multiple pathways. Alternatively, there may be a common signaling pathway modulating AP-1, NF-κB, and Nrf2 pathways. SWT has been reported by other groups with a suppressive effect on COX-2 [14], which is also involved in promoting skin carcinogenesis [29]. Since carcinogenesis involves multiple abnormal genes/pathways, using herbal medicines, such as SWT, with multiple potential targets may be superior to the agents targeting a single molecule alone. Although there are other natural products known as Nrf2 activators or AP-1/NF-κB inhibitors, SWT provides a good option as a chemopreventive agent due to its safety record. Further work is needed to identify the bioactive components and decipher which chemical components are responsible for these molecular changes. Further mechanistic studies, e.g., using animal models with gene deletion, are also needed to confirm the role of Nrf2, AP-1, and NF-κB in SWT’s chemopreventive activity. Additional investigation is needed to identify which component(s) in the AP-1 and NF-κB signal transduction pathways are involved in activity of SWT. Both AP-1 and NF-κB are transcriptional factors playing a crucial role in the regulation of cell proliferation and transformation. The present report only conducted a pilot study using luciferase assay to examine the promoter activity of AP-1 and NF-κB. Among the common downstream effector genes of AP-1 and NF-κB, we used PCNA, which is regulated by both AP-1 and NF-κB. PCNA protein level was high in the DMBA-treated, but was reduced by SWT treatment in mouse skin (Figure 4). This result confirms the role of AP-1 and NF-κB mediating SWT’s cancer preventive effect. It also remains to be determined whether SWT is able to prevent other types of cancer that are caused by environmental ROS and cellular oxidative stress.

Taken together, based on these in vitro and in vivo efficacy studies, we demonstrated the scientific evidence for SWT, one of the most popular herbal medicine formulas for women’s health, can be used for skin cancer prevention. The preliminary mechanistic studies revealed several potential molecular targets although they may not be the direct targets that the active components bind to. Obviously further studies are needed to decipher the active components for observed effect in vitro and in vivo. We predict that the chemopreventive activity of SWT should not be limited to skin cancer, but with a broader application for other types of cancer which involve ROS-related carcinogenesis.

## Figures and Tables

**Table 1:** Mutagenic activities and antimutagenic activities of SWT with or without S9.

Group	Dose (μg/plate)	−S9		+S9	
		Count	Inhibition (%)	Count	Inhibition (%)
Negative	-	132 ± 6	-	130 ± 25	-
Positive	10	3547 ± 1086	-	1409 ± 595	-
SWT	1250	125 ± 14	-	136 ± 3	-
SWT	2500	138 ± 13	-	123 ± 3	-
SWT	5000	141 ± 6	-	131 ± 6	-
SWT + Positive	1250 + 10	2688 ± 296	24.2	1246 ± 239	11.6
SWT + Positive	2500 + 10	2669 ± 282	23.9	1141 ± 129	19.0
SWT + Positive	5000 + 10	2987 ± 599	15.8	639 ± 147	54.6 **

Negative control: DMSO; Positive control: 2-Nitrofluorene (−S9); DMBA (+S9). **: significance compared to positive control group at *p* < 0.01 (*n* = 3).
